# Advancing Prevention of STIs by Developing Specific Serodiagnostic Targets: *Trichomonas vginalis* as a Model

**DOI:** 10.3390/ijerph17165783

**Published:** 2020-08-10

**Authors:** John F. Alderete

**Affiliations:** School of Molecular Biosciences, College of Veterinary Medicine, Washington State University, Pullman, WA 99164, USA; alderete@wsu.edu

**Keywords:** diagnostic, diagnostic targets, ELISA-enzyme linked immunosorbent assay, epitopes, immunogens, sera, serodiagnosis, sexually transmitted infections, *Trichomonas vaginalis*

## Abstract

Point-of-Care (POC) serum antibody screening of large cohorts of women and men at risk for the sexually transmitted infection (STI) caused by *Trichomonas vaginalis* requires the availability of targets with high specificity. Such targets should comprise epitopes unique to *T. vaginalis* immunogenic proteins detected by sera of women and men patients with trichomonosis but not uninfected controls. Three enzymes to which patients make serum IgG antibody were identified as fructose-1,6-bisphosphate aldolase (A), α-enolase (E), and glyceraldehyde-3-phosphate dehydrogenase (G). Epitopes within these proteins were identified that had no sequence identity to enzymes of humans and other pathogens. Therefore, I constructed a chimeric recombinant String-Of-Epitopes (SOE) protein consisting of 15-mer peptides, within which are the epitopes of A, E, and G. This novel protein of ~36-kD is comprised of two epitopes of A, ten epitopes of E, and seven epitopes of G (AEG::SOE2). The AEG::SOE2 protein was detected both by immunoblot and by enzyme-linked immunosorbent assay (ELISA) using highly reactive sera of women and men but not negative serum unreactive to *T. vaginalis* proteins. Finally, AEG::SOE2 was found to be immunogenic, as evidenced by serum IgG from immunized mice. I discuss how this approach is important in relation to infectious disease diagnostic targets for detection of serum IgG antibody in exposed and/or infected individuals and how such novel targets may have potential as subunit vaccine candidates against microbial pathogens.

## 1. Introduction

*Trichomonas vaginalis* causes a non-viral sexually transmitted infection (STI) with adverse outcomes to infected women [1,2]. This STI is highly prevalent [3,4,5], and persistence within individuals may be due to the asymptomatic nature of infection. It is accepted that male partners of infected women with trichomonosis become infected. The organism and *T. vaginalis* DNA have been detected in hyperplastic prostate tissue [6,7], and there remains the possibility of a link between seropositivity to *T. vaginalis* in relation to prostate cancer (PCa) development [8,9,10]. More recently, a gene-expression model for *T. vaginalis*-mediated PCa was proposed [11], and other studies lend support to this hypothesis [6,7,12,13,14,15].

A rapid, inexpensive and specific serodiagnostic that could be used for screening large cohorts of at-risk individuals is desirable. A lateral flow, immunochromatographic Point-of-Care (POC) diagnostic (OSOM^TM^ Trichomonas Rapid Test, Sekisui Diagnostics, Lexington, MA, USA) for rapid detection of active trichomonosis in women was invented in my laboratory [16]. Although the test meets criteria of being inexpensive, simple, rapid, and highly sensitive and specific, drawbacks of this test are that it is invasive for women, requiring a vaginal swab for obtaining sample, and the POC test fails to detect the specific parasite protein in the urine of male patients. Although there are numerous reports of accurate nucleic acid amplification-based tests [17,18,19], these tests are neither compatible for large scale screening in non-sterile settings nor are suitable for use in community-based clinics and at under-developed countries.

It is acknowledged that advancing the prevention of STIs in general will require specific and sensitive POC tests [20]. Such POC tests should be rapid, inexpensive, and highly dependable for serum IgG antibody detection that can be employed for broad screening of populations regardless of geographic setting. POC diagnostics are needed for surveillance of the global burden of STIs in both developed and undeveloped countries. In the case of *T. vaginalis*, surveillance is necessary among sexually active populations [20], reinforcing the view that development of a serum-antibody POC test would advance the reproductive health of at-risk women and men. Control and even elimination of *T. vaginalis* and other STIs requires an approach and method for the development of highly specific serodiagnostic targets. In this report, I provide an approach for the identification and development of serodiagnostic targets using *Trichomonas vaginalis* as a model.

As infection by *T. vaginalis* results in an IgG response [8,9,10,11,21]; I hypothesize that an approach can be developed that will lead to the synthesis of a protein for detection of serum IgG to *T. vaginalis*. Using *T. vaginalis* as a model, I present the concept that a novel, chimeric protein comprised of a String-Of-Epitopes (SOE) can be synthesized as a serodiagnostic target. My laboratory has previously determined that women and men patients make serum IgG antibody to numerous *T. vaginalis* immunogenic proteins, including the enzymes fructose-1,6-bisphosphate aldolase (referred to as A), α-enolase (E), and glyceraldehyde-3-phosphate dehydrogenase (G) [21,22,23]. Epitope mapping of these proteins with women and men patient sera identified epitopes unique to the trichomonad proteins [21]. This earlier report showed a proof-of-principle for the construction of a novel recombinant chimeric protein, called AEG::SOE, with two each of the A, E, and G epitopes of the three enzymes. This earlier construct, however, failed to detect some positive sera when compared with the gold standard immunogenic truncated version of α-actinin called ACTP2 [8,9,10,24,25]. In this report I test this hypothesis and develop a stepwise approach to show that a new recombinant protein, two epitopes of A, ten epitopes of E, and seven epitopes of G (AEG::SOE2), is a serodiagnostic target equal to ACTP2. I discuss how the approach used here may advance the development of serodiagnostic targets for this and other STIs. Finally, I show that AEG::SOE2 is immunogenic in immunized mice.

## 2. Materials and Methods

### 2.1. Epitopes Unique to the T. vaginalis A, E and G Proteins

**Figure 1:**
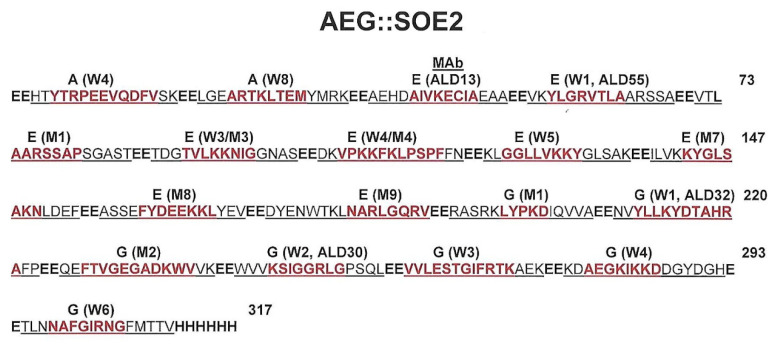
Linear amino acid sequence of the chimeric, recombinant AEG::SOE2 protein. The peptide-epitope sequences linked by EE amino acids are underlined. Epitopes within the protein–epitope sequences are colored red. The letters A, E, and G refer to amino acid sequences derived from respective proteins. The epitopes were detected by either or both pooled women (W) or men (M) sera, as indicated, and the number next to W and M represents the order the epitope was identified within the protein during epitope mapping on the SPOTs system.

The identification of immunogenic epitopes reactive to women and men patient sera was done using oligopeptides (Custom Peptide Arrays) immobilized on membranes (SPOTs system; Sigma-Aldrich Corp, St Louis, MO, USA) as recently detailed [21,24]. As before, oligopeptides of fructose-1,6-bisphosphate aldolase (A), α-enolase (E), and glyceraldehyde-3-phosphate dehydrogenase (G) were derived from GenBank^®^ accession numbers AAW78351 (A), AAK73099 (E), and AAA30325 (G). The protocols for probing of SPOTs membranes with sera were as detailed before [21]. The epitopes of A, E, and G reactive with women and men positive control sera were presented in an earlier publication [21]. Further, sequence identity analysis was performed with the enzyme homologs of human and other eukaryote and bacterial pathogens [21]. Finally, each 15-mer peptide within which the epitopes resided (Figure 1) was analyzed using the Immune Epitope Database and Analysis Resource (www.iedb.org) to show the linear nature of the peptide–epitope sequence.

### 2.2. ACT-P2, a Truncated α-Actinin Protein Used for Screening of Patient Sera

The α-actinin protein is one of the most immunogenic proteins of *T. vaginalis* [26,27] and has been shown to be a serodiagnostic target because it has no sequence identity to other known proteins in databases [24]. The truncated α-actinin protein called ACT-P2 of 558-amino acids (64.1-kDa) has been previously described [25]. ACT-P2 has been used to screen both male and female patient sera [24] and more recently to examine the relation of serum antibody in men and prostate cancer [8,9,10]. Thirteen epitopes were identified as reactive with women sera. In men, 5 of the 13 reactive epitopes were detected [24].

### 2.3. Plasmid Encoding AEG::SOE2 and ACT-P2

The DNA coding sequence for the chimeric, recombinant AEG::SOE2 protein was synthesized by GenWay Biotech, Inc (San Diego, CA, USA). The pET-23a (+) plasmid that was prepared was encoded for ampicillin (Amp ^+^) and chloramphenicol (Cam ^+^) genes. The recombinant *E. coli* BL21DE3 cells were used to synthesize the protein. The description of ACT-P2 in *E. coli* has been described [24]. Both recombinant proteins have hexa-histidine at the carboxy terminus for purification [21,25].

### 2.4. Recombinant Proteins

**Figure 2:**
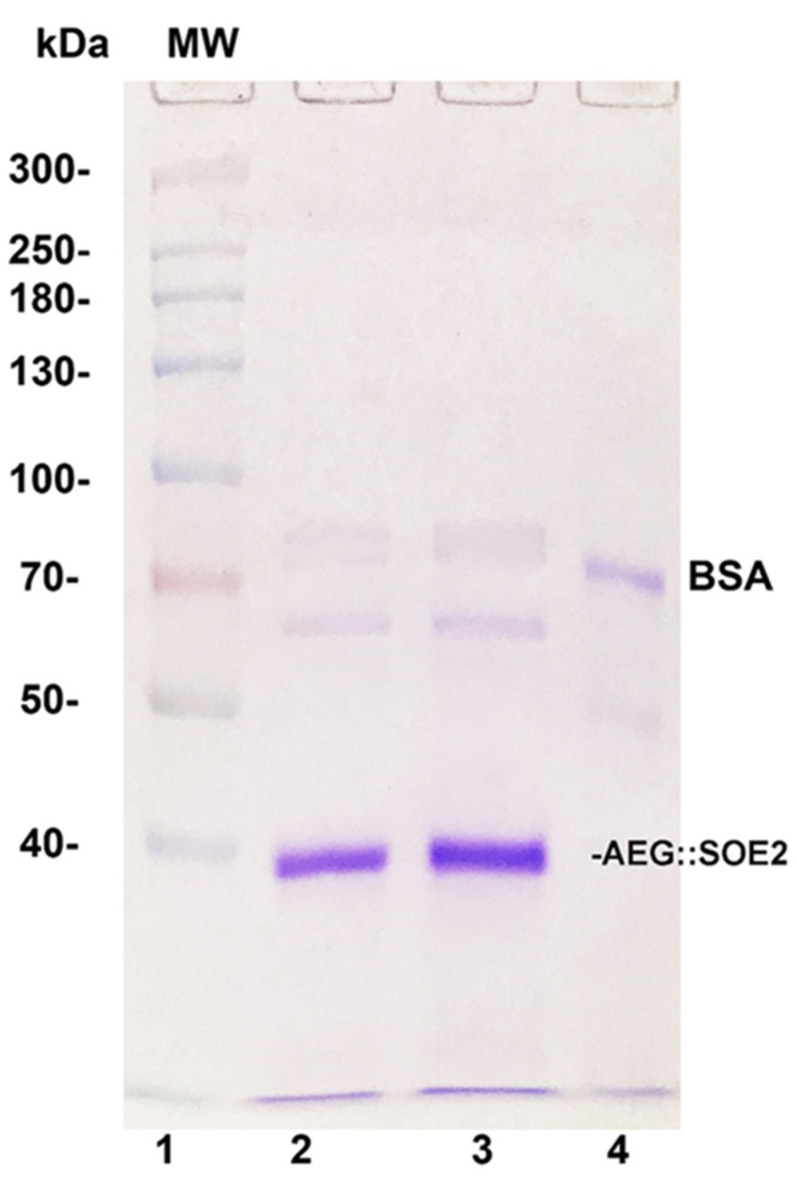
Sodium dodecyl sulfate-polyacrylamide gel electrophoresis (SDS-PAGE) in 8% acrylamide of AEG::SOE2 from two different experiments (lanes 2 and 3) after purification by Ni^2+^ NTA affinity chromatography. Lane 1 is of molecular weight (MW) standards, and numbers refer to daltons (×1000). Lane 4 is of 1µg bovine serum albumin (BSA) electrophoresed for comparative purposes.

Detailed protocols for growing bacteria on Luria Broth (LB) agar plates with either 25 µg/mL Kanamycin (ACT-P2) or 100 µg/mL Ampicillin (AEG::SOE2) have been published [25]. A starter culture of LB medium with antibiotics was inoculated with recombinant *E. coli* and grown as before [21,22,23,24] prior to inoculation of medium and induction for expression with isopropyl β-D-1-thiogalactopyranoside (IPTG) for synthesis of recombinant proteins. Purification of the recombinant proteins was as before [21,24,25]. Purified proteins were obtained by Ni^2+^-NTA superflow affinity column chromatography (Qiagen Inc., Valencia, CA, USA), and soluble AEG::SOE2 was recovered from *E. coli* lysates using the Ni^2+^-NTA column, as evidenced by Figure 2. It is noteworthy that protein was also detected within inclusion bodies, and GenWay, Inc. indicates it is able to purify AEG::SOE2 from these inclusion bodies.

### 2.5. Sera for Enzyme-Linked Immunosorbent Assay (ELISA) and Derivation of Positive/Negative (P/N) Scores

Numerous reports have described the sera from women and men used for detection of ACTP2 [21,24]. Sera were also derived from research conducted at Washington University as reported earlier [8,9,10]. Institutional Review Board (IRB) approvals for collecting and using the sera were obtained at Washington University-Saint Louis, MO as well as by the IRB at Washington State University (approval number 01058) [8,9,10]. Equally importantly, the use of sera of patients has been reported before [28,29,30], and patients were diagnosed by microscopy and positive cultures.

My laboratory has published detailed descriptions for the use of sera by ELISA for detection of proteins, and identical materials and methods published earlier were used in this study to compare reactivities with ACTP2 and AEG::SOE2 [8,9,10,21,25]. Briefly, wells of microtiter plates prepared as before were stored at 4 °C prior to use. The processing of plates has been detailed before [25]. All washes of wells used phosphate-buffered saline (PBS), pH 7.4 containing 0.05% Tween-20 (PBS-Tween). Importantly, blocking of wells prior to addition of sera and dilutions of sera were done using a solution of 2% ELISA-grade bovine serum albumin (eBSA) (Sigma Chemical Co., St. Louis, MO, USA) prepared in PBS (eBSA-PBS). Where indicated, a 50 µL volume of undiluted hybridoma supernatant of a monoclonal antibody (MAb) or a cocktail of 25 µL for each of 4 MAbs (Figure 1) were used. Unless where indicated, all sera used for ELISA were diluted 1:25 (v/v) with eBSA-PBS.

In order to perform comparative analyses of ELISA using different targets, it was necessary to have serum standards as negative (scored as 0, 1+ and 2+) and positive (scored as 3+ and 4+) controls derived from testing ACTP2 as the gold standard for screening [8,9,10,24,25]. My laboratory previously determined the range of ELISA values for the different scores. Positive 3+ and 4+ sera detected trichomonad proteins by immunoblot, and 0, 1+ and 2+ sera did not detect proteins under the same conditions [24]. For these experiments obtaining P/N values during ELISAs to provide scores 0 to 4+, the absorbance mean ± standard deviations were as follows: the blank control with eBSA-PBS was 0.050 ± 0.002, the 0 (zero) score was 0.131 ± 0.012, the 1+ score was 0.187 ± 0.010, and the 2+ score was 0.233 ± 0.023. The scores for 3+ and 4+ for obtaining absorbance values were 0.311 ± 0.025 and 0.441 ± 0.20, respectively. All scores were derived by subtracting the average blank optical density (OD) reading.

### 2.6. Mouse Anti-AEG::SOE2 Serum and Anti-T. vaginalis Serum and ELISA for Detecting Antibody to T. vaginalis

Mouse anti-*T. vaginalis* serum was obtained by immunizing BALB/c mice as previously described [31]. Mouse anti-AEG::SOE2 serum (IMS) was derived by immunizing mice using the Washington State University Antibody Core Facility of the College of Veterinary Medicine. In this case, mice were immunized subcutaneously twice with 50 µg of purified protein (Figure 2) at 2-week intervals followed by the last booster injected into the tail vein. Prebleed normal mouse serum (NMS) was obtained prior to immunization and used as a negative control. All animals were treated humanely as governed by the Institutional Animal Use and Care Committee (IACUC number 6317) and National Institutes of Health protocols.

We also performed a whole cell ELISA for detecting antibody using microtiter wells coated with trichomonads. Parasites at logarithmic growth were washed three times with PBS, and a 50 µL suspension containing 1.25 × 10^5^ organisms was added to individual wells of microtiter plates. After drying at 37 °C, 50 µL of 95% ethanol was added as fixative and wells were allowed to dry. Both protein-and trichomonad-coated wells were washed three times with PBS-Tween followed by blocking with 200 µL of eBSA-PBS. The remaining standard protocol is as previously reported elsewhere [8,9,10,21,24,25].

### 2.7. Reproducibility

All experiments were performed at least three times under identical conditions. ELISAs on microtiter plates coated with proteins or trichomonads were done in quadruplicate unless otherwise indicated, and means and standard deviations were calculated. All statistical analyses were conducted with RStudio (Version 1.2.5033; RStudio, Inc.: Vienna, Austria), and figures were made in Prism (Version 8.4.3; GraphPad, LLC: San Diego, CA, USA). T-tests were used to examine differences in absorbance levels for ACTP2 and AEG::SOE2 for mouse sera, negative and positive human sera, and monoclonal antibodies. Statistical significance was defined as a *p*-value less than 0.05.

## 3. Results

### 3.1. Epitopes of A, E, and G Unique to T. vaginalis and the AEG::SOE2 Protein Sequence

Epitope mapping revealed that pooled women and men positive serum recognized a total of 12 epitopes of A, 18 epitopes of E, and 19 epitopes of G, for a total of 49 epitopes for the three proteins [21]. Table 1 shows there were only 2 of 12, 9 of 18, and 7 of 19 epitopes unique to the *T. vaginalis* A, E, and G proteins, respectively, that had no sequence identity with other bacterial, fungal, parasite, and human sequences [21]. The red underlined amino acid sequences are the epitopes detected by IgG antibody in the SPOTS system used for screening. Not surprisingly, the epitopes that were not unique to *T. vaginalis* proteins had identity, albeit to different degrees, with protein sequences of enzymes of other bacterial, fungal, parasite, and human proteins (data not shown).

Figure 1 presents the recombinant protein amino acid sequence representing the peptides containing the unique epitopes shown above in Table 1, and each peptide sequence was linked with two glutamic acid residues. The reactivity with women (W) and/or men (M) sera is shown above the red epitope amino acid sequences. The protein has a M_r_ of 35,896.31 daltons and pI of 5.05. Overall, there are 8 and 11 epitopes detected by positive women and men sera, respectively. The four monoclonal antibodies (MAbs), labeled ALD 13, ALD 55, ALD 32, and ALD 30, are reactive with the epitopes, as indicated, and these MAbs have been previously characterized [21].

### 3.2. Purification of Recombinant AEG::SOE2

Figure 2 shows the Coomassie-blue-stained gel after sodium dodecyl sulfate-polyacrylamide gel electrophoresis (SDS-PAGE) of purified AEG::SOE2 from two different experiments (lanes 2 and 3). The relative mobility of the protein was compared with molecular weight standards (lane 1) and is consistent with the expected size of ~35.9-kDa. Lane 4 shows the stained band of 1 µg of BSA for comparison.

### 3.3. ELISA Using Different Amounts of AEG::SOE2-Coated Wells Probed with Different Antibodies

**Figure 3:**
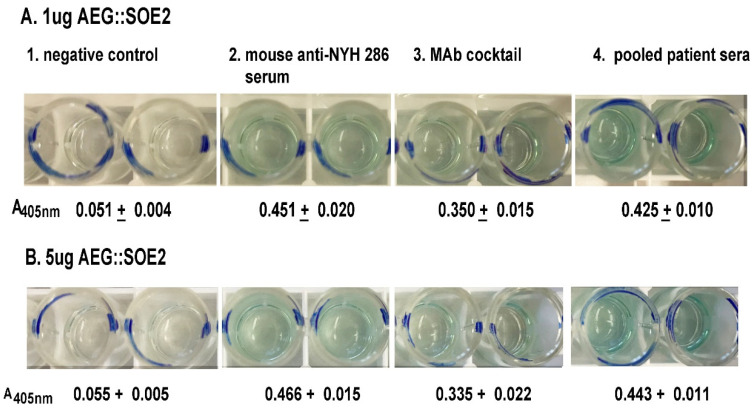
ELISA for detection of AEG::SOE2 at 1µg (**A**) and 5µg (**B**) AEG::SOE2 immobilized onto individual wells of 96-well microtiter plates. The negative controls were wells incubated with five different pooled negative sera each from women and men. This sera were shown previously to have no reactivity with any *T. vaginalis* proteins by immunoblot [24] (wells numbered 1). Wells with the different concentrations of AEG::SOE2 protein were incubated with mouse anti-*T. vaginalis* serum [31] (wells numbered 2), a cocktail of MAbs reactive with AEG::SOE2 epitopes as shown in Figure 1 (wells numbered 3), and with five different pooled positive women and men sera (wells numbered 4). The negative and positive pooled women and men sera have been previously reported [26]. Values were obtained by absorbance at 405 nm. As expected, a negative control irrelevant MAb to α-actinin called HA423 [24] was unreactive to AEG::SOE2 by ELISA, as shown in Figure 4. The ELISA was repeated on four different times with similar results.

ELISA was then performed to determine the amount of AEG::SOE2 immobilized onto microtiter wells detected by pooled positive patient sera, mouse anti-*T. vaginalis* serum [31], and a cocktail of MAbs reactive to the protein (Figure 1). As seen in Figure 3, 1µg (**A**) and 5 µg (**B**) of protein on wells were detected by the mouse antiserum at a 1:100 dilution (wells numbered 2), the cocktail of IgG_1_ monoclonal antibodies (MAbs) (wells numbered 3, 25 µL of hybridoma supernatant of each MAb), and pooled patient sera at a 1:25 dilution (wells numbered 4). Not surprisingly, the protein (Figure 2) was also detected by immunoblot with these antibody reagents. There was no detection of AEG::SOE2 even at 10 µg amounts using negative pooled sera of women and men (wells number 1). An irrelevant MAb to the actinin protein called HA423 [24], as shown also in Table 2, was unreactive to AEG::SOE2. One microgram amounts of AEG::SOE2 were chosen as the standard amount for ELISA as less than 1µg coated onto wells gave mixed results with patient sera and MAbs. The 1:25 dilution for patient sera was shown previously to be ideal for ELISA with 1µg of protein [8,9,10,21,24,25].

### 3.4. ELISA Comparing ACT-P2 and AEG::SOE2 with Different Antibodies

**Figure 4:**
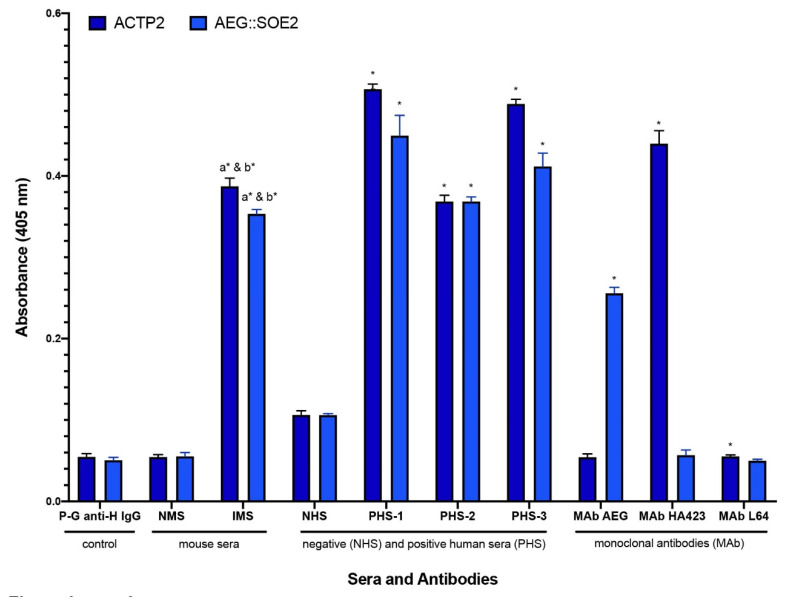
ELISA comparing negative (NHS) and positive women and men sera (PHS) and prebleed, normal mouse serum (NMS), and immunized mouse anti-*T. vaginalis* serum (IMS), for detection of IgG antibody to ACT-P2 (dark blue) and AEG::SOE2 (light blue). Bars represent means and standard deviations that were calculated for the average of all ELISA performed (n = 8). The secondary peroxidase-conjugated goat anti-human IgG (Fc fraction; labeled P-G anti-H IgG) is the secondary antibody used for ELISA for detecting human antibody and gave values equal to the use of 2% eBSA-PBS alone as a negative control. NMS sera gave values equivalent to secondary antibody alone. The pooled negative human sera (NHS) lacking reactivity to trichomonad proteins [24,25] represented five different women and five different men sera. The three pooled positive human sera (PHS) that detect trichomonad proteins represented either five women sera (number 1), five men sera (number 2), or five combined women and men sera (number 3). Independent t-tests were used to compare the mean absorbance levels for each target protein to its corresponding control. This included IMS vs. P-G anti-H IgG (a), IMS vs NMS (b), PHS-1 vs NHS, PHS-2 vs NHS, and PHS-3 vs NHS. The MAb cocktail mix is comprised of equal volumes (25 µL) of hybridoma supernatants of the four MAbs to the epitopes of the recombinant AEG::SOE2 (Figure 2). HA423 is an MAb directed to ACTP2 and is reactive with ACTP2 but not AEG::SOE2 [21,24,25]. MAb L64 is an irrelevant control antibody that reacts with a cytoplasmic protein of *T. vaginalis* [22]. All monoclonal antibodies are of the IgG_1_ isotype. Lastly, differences in mean absorbance levels for each monoclonal antibody (i.e., cocktail MAb AEG, MAb HA423, and MAb L64) by protein type (i.e., ACTP2, AEG::SOE2) were examined. Absorbance values were obtained at 405nm. The means and standard deviations statistical significance is denoted as asterisks (*) and found to be *p* < 0.001.

We now compared AEG::SOE2 by ELISA with the gold standard ACTP2 for detection of IgG. Figure 4 compares ELISA values for negative human sera (NHS) versus positive women and men sera (PHS) characterized previously [21,24,25]. The pooled PHS known to have antibody to trichomonad proteins were five each of women sera (PHS-1), men sera (PHS-2), or combined women and men sera (PHS-3). ELISA results show statistically significant higher absorbance values for PHS to both ACTP2 and AEG::SOE2 when compared with NHS of five different women and five different men sera that lacked reactivity to trichomonad proteins by immunoblot. Additionally, sera of mice immunized with *T. vaginalis* (IMS) gave statistically significant higher values to both ACTP2 and AEG::SOE2 when compared with both prebleed, normal mouse sera (NMS), and secondary peroxidase-conjugated goat anti-human IgG alone (P-G anti-H IgG). Finally, and not unexpectedly, the MAbs to AEG::SOE2 (Figure 2) gave statistically significant higher values with AEG::SOE2 but not ACTP2, and the MAb HA423 to ACTP2 was unreactive with AEG::SOE2 compared to statistically significant values with ACTP2. MAb L64 is a trichomonad cytoplasmic protein and was unreactive to both ACTP2 and AEG::SOE2.

### 3.5. Different ELISA Experiments Testing Women and Men Sera of Different 0 to 4+ Scores

Table 2 shows results of quadruplicate testing using up to ten individual women and men sera scored from 0 to 4+ based on reactivity to ACT-P2. Only 3+ and 4+ sera have been shown to have IgG antibody to trichomonad proteins [21,24,25]. Not unexpectedly, the negative women (W) and men (M) sera remained 0 to 2+ for ACT-P2 and AEG::SOE2. In contrast, and as shown within the red boxes, all of the 3+ and 4+ positive sera gave reproducible high reactions for both proteins.

**Figure 5:**
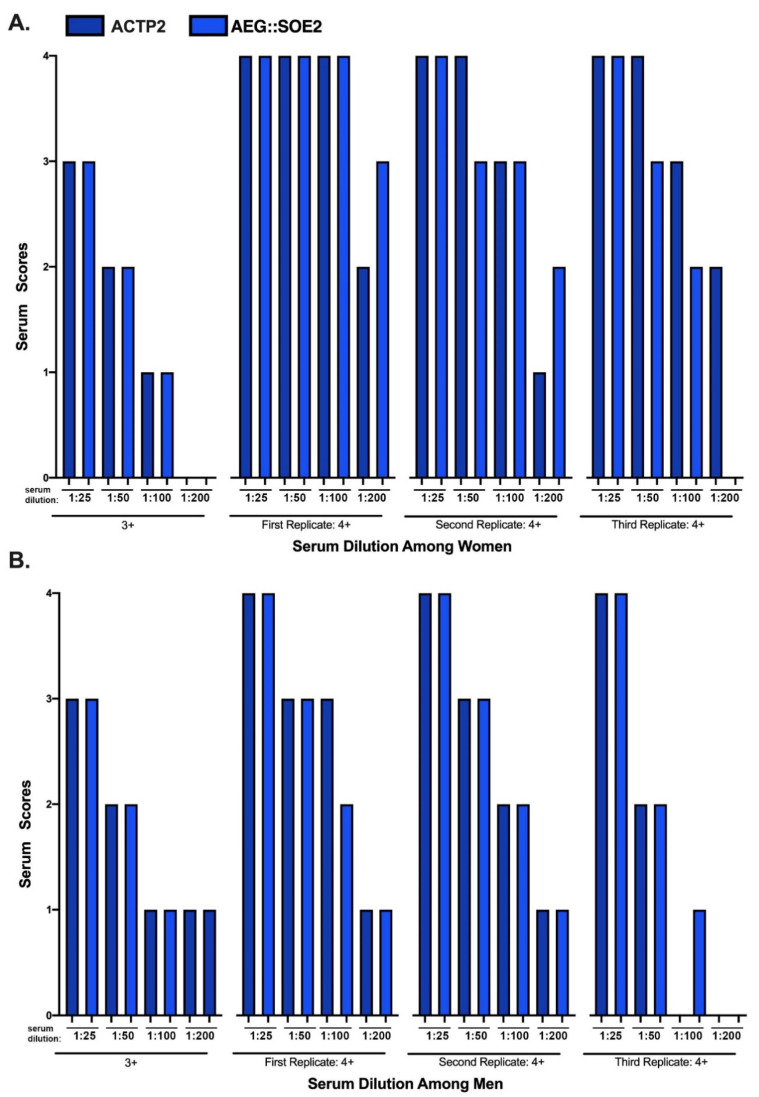
ELISA of women and men sera of 3+ and 4+ scores at different dilutions. The sera designations for 3+ and 4+ scores following ELISA using standards as controls is as described above for Table 2. This scoring enables the comparative examination of dilutions with eBSA-PBS on the relative reactivities against the target ACT-P2 and ACT::SOE2 proteins. Each replicate consists of pooled different positive sera (n = 10) of women and men.

For clarification of what the scores signify in terms of the mean and standard deviation (SD) absorbance readings for Table 2 and for Figure 5 and Table 3 below, the blank control with eBSA-PBS only was 0.050 ± 0.002. The mean and SD for sera giving different scores were as follows: sera with a 0 (zero) score was 0.131 ± 0.012, sera with a 1+ score was 0.187 ± 0.010, and the sera with a 2+ score was 0.233 ± 0.023. The sera with scores of 3+ and 4+ were 0.311 ± 0.025 and 0.441 ± 0.20, respectively. All scores were derived by subtracting from the average blank OD reading of 0.050.

### 3.6. ELISA of Women and Men Sera of 3+ and 4+ Scores at Different Dilutions

Figure 5 compares the positive 3+ and 4+ sera reactivities toward ACTP2 (dark blue) and AEG::SOE2 (light blue) using women and men sera at different dilutions. Not shown is that the 0 to 2+ sera remained negative at all dilutions for both target proteins. For both ACTP2 and AEG::SOE2, the 3+ women (part A) and men (part B) sera became negative (2+ or lower) at dilutions of 1:50. Part A shows that the three replicates of the 4+ women sera were positive for ACTP2 at 1:100 dilutions. Only the first and second replicates were positive for AEG::SOE2 at 1:100 dilutions, but the third replicate sera were positive only up to the 1:50 dilution. Interestingly, the first replicate 4+ sera remained positive for AEG::SOE2 at 1:200 dilution. Part B shows that the first and second replicates of 4+ men sera were positive for ACTP2 at the 1:50 dilution, and only the first replicate sera was positive at the 1:100 dilution. For AEG::SOE2, only the first and second replicates remained positive at the 1:50 dilution. Overall, the data suggest that the 4+ sera of both women and men have higher titers of IgG than the 3+ sera for both target proteins.

### 3.7. ELISA Using ACT-P2 and AEG::SOE2 as Targets with 0 to 4+ Individual Sera

Finally, I randomly selected 42 each of sera of women and men with different scores for side-by-side evaluation. Table 4 presents results showing that, except for a few samples, there was almost 100% agreement for 3+ and 4+ positive sera for both ACT-P2 and AEG::SOE2. The score of 3++ had average and standard deviations much higher than the value for 3+ given above for Table 2. Likewise, there was almost 100% agreement for the 0 to 2+ negative sera. Interestingly, there were four 3+ sera that were reactive with ACT-P2 but not AEG::SOE2. Similarly, there were four 3+ sera that detected AEG::SOE2 but not ACT-P2. One possible explanation for these latter results is that the 3+ sera may be borderline negative, and this will require testing to determine whether or not there is IgG antibody that detects trichomonad proteins, such as by immunoblot, as before [21,24].

### 3.8. AEG::SOE2 is Immunogenic, and Anti-AEG::SOE2 Serum IgG Antibody Detects T. vaginalis Organisms Immobilized onto Microtiter Wells by ELISA

Finally, we wanted to examine whether mice immunized with AEG::SOE2 produced IgG antibodies. We compared mouse anti-AEG::SOE2 serum with mouse anti-*T vaginalis* serum [29] in individual wells of microtiter plates coated with fixed trichomonads. As shown in Table 4, both antisera gave ELISA readings greater than secondary peroxidase-conjugated goat anti-mouse IgG alone and prebleed normal mouse serum (NMS). Likewise, a cocktail of hybridoma supernatants of the MAbs reactive with AEG::SOE2 epitopes and MAb ALD30A alone (Figure 1) gave higher values compared to controls. The MAb L64 of the same isotype as the MAbs to AEG::SOE2 was used as another negative control. Furthermore, that MAbs to E and G react with trichomonads in this whole cell ELISA further supports earlier work that these metabolic enzymes are on the surface of *T. vaginalis* [23,24].

## 4. Discussion

In this study, I use an approach to extend an earlier published work [21] and show the synthesis of a larger novel, chimeric String-Of-Epitopes (SOE) protein called AEG::SOE2 with additional epitopes of A, E, and G. This AEG::SOE2 protein possesses the same high specificity and sensitivity as ACT-P2, the gold-standard target for *T. vaginalis* seropositivity (Figure 4 and Table 2; Table 3). These data indicate that AEG::SOE2 may be a target for a rapid, accurate, and cost-effective POC test. Such a test would allow for screening of individuals with active *T. vaginalis* infection or permit identification of those previously exposed to the organism. Another reason for moving toward a serum-based diagnostic is the demonstration of positive IgG seroconversion in relation to *T. vaginalis* and PCa development and progression [8,9,10,11]. The next step now appears to be development of a platform incorporating AEG::SOE2 in order to demonstrate a POC diagnostic for broad application of *T. vaginalis* surveillance.

As discussed recently [25], little is known of the temporal nature and duration of the serum IgG antibody responses among patients after infection with *T. vaginalis* and after diagnosis and cure. Such a POC test would also permit the medical community to understand the specific IgG response to the parasite in relation to active and/or past infections. My laboratory showed the short-lived nature of both serum and vaginal IgG to trichomonad cysteine proteinases after treatment of patients [28,29]. My laboratory also reported that IgG to a 230-kDa trichomonad protein was still evident in vaginal washes of patients at 4-weeks post cure [30]. I believe that these earlier findings support the view that a serodiagnostic test is necessary in order to understand the antibody responses of infected individuals. The availability of proteins unique to *T. vaginalis,* like α-actinin [24] and AEG::SOE2, provides the opportunity to elucidate the extent and nature of the antibody response issues in the future. The data (Figure 4 and Table 2) presented here show that AEG::SOE2 is equivalent to ACTP2 in serum IgG immunoreactivity. The presence of serum IgG antibody to epitopes unique to *T. vaginalis* proteins further reinforces the legitimacy of the approach taken here for identifying a specific and novel target for *T. vaginalis,* and although speculative, it may be possible to develop tests for other STI microbial pathogens and additional infectious diseases using this approach.

These novel, chimeric SOE proteins are comprised of immunogenic epitopes unique to the microbial pathogen of interest and, in this case, *T. vaginalis*. It is intriguing to consider that these SOE proteins may have efficacy as vaccines. At present, there is no evidence of immune protection against *T. vaginalis* despite the presence of both serum and vaginal antibody responses among patients with trichomonosis [28,29,30]. The many trichomonad proteases that degrade immunoglobulins may be a reason for immune evasion [29]. Reports have proposed that whole *T. vaginalis* organisms or lysates may be used as vaccines for *T. vaginalis* [32,33]. I argue against using *T. vaginalis* organisms or lysate as vaccines. One reason is that serum IgG antibody is made to epitopes of these enzymes, which in fact have amino acid sequence identities with human enzymes (referred to as trichomonad non-unique epitopes) [21]. In other words, as mentioned for Table 1, 10 of 12 epitopes of A, 9 of 18 epitopes of E, and 12 of 19 epitopes of G had sequence identity to human, bacterial, parasite, and fungal enzyme proteins. This is an important finding in and of itself that should be considered when studying host antibody responses to microbial pathogens. Whether antibodies to these trichomonad, non-unique epitopes common to human proteins mediate auto-immune reactions and, therefore, possible tissue damage is presently unknown. I believe that this issue must be considered within the framework of pathogenesis of trichomonosis and also for other infectious diseases. Indeed, it has been shown that human serum antibody to α–enolase of group A streptococcus cross-reacts with host tissues [34]. Thus, the approach described here may circumvent potential immune-crossreactive problems posed by using whole cell or lysate vaccines.

Another reason against whole organisms and lysates as vaccines is that we now know that *T. vaginalis* acquires onto its surface numerous host serum proteins [22,23,35,36,37,38]. The coating of the parasite surface with host proteins [36,38] may represent yet another mechanism for parasite evasion of immune-antibody responses. Further, the *T. vaginalis* surface-associated E and G metabolic enzymes are ligands that bind host proteins, such as plasminogen, fibronectin, collagen, and laminin [22,23]. As these host proteins may play a role in pathogenesis, the proteins are referred to as host-pathogenicity factors [22,23,35,36,37,38]. It is possible that host proteins on *T. vaginalis* may have altered structures exposing epitopes to host antibody responses, creating possible auto-antibodies and adverse reactions with tissues. This possibility deserves more attention in host–parasite interactions.

Of interest is that trichomonad lysates were recently compared with α-actinin by ELISA for IgG reactivity [39], and both were found to be equivalent in serum IgG detection. Here, too, I argue that lysates are inappropriate diagnostic targets for the same reasons mentioned above. Seropositive reactions may be due, in part, to IgG antibody responses to metabolic enzyme epitopes common to other pathogens. In this scenario, infections by other bacterial, parasite, and fungal pathogens may induce IgG not only to the epitopes of the enzymes A, E, and G used here but also to epitopes of other proteins that are immuno-crossreactive with *T. vaginalis* proteins. This conclusion has merit based on the findings presented here. This, then, would lead to false-positive reactions for this STI. Therefore, future serodiagnostic targets for this and other infectious diseases must have specific epitopes that are unique to the pathogen causing the disease. These concerns are relevant to the development of effective infectious disease diagnostics and vaccines and are also important considerations for surveillance and interventions of STIs and infectious diseases [40].

Finally, I have shown that the chimeric AEG::SOE2 protein is immunogenic, as evidenced by IgG antibody made by immunized mice (Table 4). Importantly, that anti-AEG::SOE2 serum detects whole organisms reaffirms the surface location of these proteins on *T. vaginalis* [22,23]. More importantly, multi-epitope constructs like AEG::SOE2 may be tested as a vaccine candidate for *T. vaginalis*, and this may be verified as was recently shown by others for α-actinin [41]. I believe that the approach described here may lead to future specific diagnostic targets and that such targets, comprised of immunogenic epitopes unique to the pathogen of interest, can be possible effective subunit vaccine candidates.

## 5. Conclusions

A stepwise approach is presented that may have applicability for infectious diseases by producing a unique and specific serodiagnostic target to an infectious agent, and such SOE proteins can be tested for specificity as a diagnostic target. *Trichomonas vaginalis* was used as a model to test the viability of the approach. This approach includes i) identification of immunogenic surface proteins; ii) epitope mapping; iii) selection of epitopes with amino acid sequences unique to the trichomonad proteins; and iv) construction of a hybrid String-Of-Epitopes AEG::SOE2 protein comprised of A, E, and G epitopes. Finally, because of the highly immunogenic nature of the epitopes, as evidenced by patients’ serum IgG reactivities (Figure 3; Figure 4 and Table 3), the fact that AEG::SOE2 itself elicited IgG antibodies via immunization (Table 4), and the fact that the epitopes have no identity to other known proteins in databanks, this novel SOE protein of *T. vaginalis* and SOE proteins of infectious agents in general may have potential vaccine applicability.

## 6. Patent

J.F. Alderete. Strings of Epitopes Useful in Diagnosing and Eliciting Immune Responses to Sexually Transmitted Infections. No. 9910042, 5 March, 2018.

J.F. Alderete. Strings of Epitopes Useful in Diagnosing and Eliciting Immune Responses to Sexually Transmitted Infections. No. 10386369, 20 August, 2019.

## Figures and Tables

**Table 1:** The amino acid sequences containing the immunogenic epitopes (red underlined) of two epitopes of A, ten epitopes of E, and seven epitopes of G (AEG::SOE2) protein **^ǂ^**.

		Proteins	
No.	A	E	G
1	HTYTRPEEVODFVSK	AEHDAIVKECIAEEAA	RACRKLYPKDIQVVA
2	LGEARTKLTEMYMRK	VKYLGRVTLAARSSA	NVYLLKYDTAHRAFP
3		VTLAARSSAPSGAST	QEFTVGEGADKWVVK
4		TDGTVLKKNIGGNAC	WVVKSIGGRLGPSQL
5		DKVPKKFKLPSPFFN	VVLESTGIFRTKAEK
6		KLGGLLVKKYGLSAK	KDAEGKIKKDDGYDGH
7		ILVKKYGLSAKNLDEF	TLNNAFGIRNGFMTTV
8		ASSEFYDEEKKLYEV	
9		DYENWTKLNARLGQRV	

**ǂ** AEG::SOE2 refers to the chimeric protein derived from epitopes unique to the *T. vaginalis* proteins fructose-1,6-bisphosphate aldolase (A), α-enolase (E), and glyceraldehyde-3-phosphate dehydrogenase (G), as described in Figure 1.

**Table 2:** Testing by ELISA of women and men sera of different positive to negative (P/N) **^‡‡^** 0 to 4+ scores.

**Expt**	**Serum Reaction Scores Against ACTP2:**													
	**0^‡‡^**	**0**	**1+**	**1+**	**2+**	**2+**	**3+**	**3+**	**4+**	**4+**	**4+**	**4+**	**4+**	**4+**
	**W**	**M**	**W**	**M**	**W**	**M**	**W**	**M**	**W**	**M**	**W**	**M**	**W**	**M**
**expt 1**	0	0	1	1	2	2	3	3	4	4	4	4	4	4
**expt 2**	0	0	1	1	2	2	3	3	4	4	4	4	4	4
**expt 3**	0	0	1	1	2	2	3	3	4	4	4	4	4	4
**expt 4**	0	0	1	1	2	2	3	3	4	4	4	4	4	4
**Expt**	**Serum Reaction Scores Against AEG::SOE2:**													
	**0**	**0**	**1+**	**1+**	**2+**	**2+**	**3+**	**3+**	**4+**	**4+**	**4+**	**4+**	**4+**	**4+**
	**W**	**M**	**W**	**M**	**W**	**M**	**W**	**M**	**W**	**M**	**W**	**M**	**W**	**M**
**expt 1**	0	0	1	1	2	2	3	3	4	4	4	4	4	4
**expt 2**	0	0	1	1	2	2	3	3	4	4	4	4	4	4
**expt 3**	0	0	1	1	2	2	3	3	4	4	4	4	4	4
**expt 4**	0	0	1	1	2	2	3	3	4	4	4	4	4	4

**^‡‡^** Serum designations refer to the 0–4+ scoring of the sera following ELISA using standards as controls as described above. This scoring enables the examination of the relative reactivities against the ACT-P2 and ACT::SOE2 proteins. The red scores represent sera with reactivities to trichomonad proteins by immunoblot.

**Table 3:** Reactivities by ELISA using ACTP2 comparing with AEG::SOE2 as targets.

No.^‡^	Scores ^#^	Scores	No.	Scores	Scores	No.	Scores	Scores	No.	Scores	Scores
	AEG::SOE2	ACTP2		AEG::SOE2	ACTP2		AEG::SOE2	ACTP2		AEG::SOE2	ACTP2
**1**	0	0	**22**	4+	3++	**43**	4+	4+	**64**	3+	2
**2**	4+	4+	**23**	3+	3+	**44**	3+	3+	**65**	4+	3++
**3**	3+	2++	**24**	4+	4+	**45**	3+	3+	**66**	2+	2+
**4**	1+	2+	**25**	4+	4+	**46**	4+	3+	**67**	3+	3++
**5**	2+	3+	**26**	3++	3+	**47**	2+	2+	**68**	2+	2+
**6**	2+	2+	**27**	4+	3++	**48**	3++	3+	**69**	2+	2+
**7**	3+	4+	**28**	4+	3++	**49**	3+	3+	**70**	3+	3++
**8**	2+	2+	**29**	2+	2+	**50**	2+	3+	**71**	1+	1+
**9**	2+	2+	**30**	1+	1+	**51**	3+	2+	**72**	0	0
**10**	4+	4+	**31**	1+	1+	**52**	4+	3++	**73**	1+	1+
**11**	4+	3++	**32**	1+	2+	**53**	3+	2+	**74**	0	0
**12**	2+	3+	**33**	4+	3++	**54**	3+	1+	**75**	2+	3+
**13**	1+		**34**	4+	3+	**55**	3++	3+	**76**	0	1+
**14**	4+	4+	**35**	3++	4+	**56**	2+	2+	**77**	4+	4+
**15**	0	0	**36**	3+	2+	**57**	3+	3++	**78**	3++	3++
**16**	2+	2+	**37**	3+	3+	**58**	4+	4+	**79**	3+	3+
**17**	4+	4+	**38**	3+	2+	**59**	3+	3+	**80**	3+	3+
**18**	3+	3+	**39**	3++	3+	**60**	4+	4+	**81**	4+	4+
**19**	4+	3++	**40**	1+	1+	**61**	4+	3++	**82**	3+	3+
**20**	3++	4+	**41**	2+	1+	**62**	3+	3+	**83**	4+	4+
**21**	4+	4+	**42**	3+	3+	**63**	3+	3+	**84**	4+	4+

**^‡^** Refers to the number of the serum sample used for the ELISA comparisons of AEG::SOE2 and ACTP2. ^#^ All experiments were performed using quadruplicate wells. Agreement for 3+ and 4+ sera for both ACTP2 and AEG::SOE2 is denoted in yellow. The four 3+ sera that were reactive with ACTP2 but not AEG::SOE2 are denoted in green, and the four 3+ sera positive for AEG::SOE2 but not ACTP2 are denoted in blue.

**Table 4:** Whole cell ELISA for detection of anti-*T. vaginalis* mouse serum IgG antibody and monoclonal antibody (MAb).

	Experiment No. (Mean ± SD)		
Antibodies *	1	2	3
goat anti-mouse IgG alone	0.075 ± 0.001 ^‡^	0.123 ± 0.005	0.130 ± 0.015
NMS (1:10)	0.160 ± 0.001	0.157 ± 0.002	0.140 ± 0.010
anti-*T. vaginalis serum* (1:10)	ND ^‡‡^	0.364 ± 0.005	0.455 ± 0.005
anti-*T. vaginalis serum* (1:100)	0.345 ± 0.005	ND	0.432 ± 0.001
anti-AEG::SOE2 serum (1:10)	0.225 ± 0.002	ND	ND
anti-AEG::SOE2 serum (1:10)	0.207 ±0.002	0.305 ± 0.005	0.190 ± 0.005
MAb cocktail (1:1:1:1, v/v)	0.275 ± 0.005	0.218 ± 0.005	0.228 ± 0.005
MAb ALD30A	0.335 ± 0.010	0.220 ± 0.010	0.253 ± 0.008
MAb L64	0.055, 0.055	0.051, 0.052	0.051, 0.050

* The peroxidase-conjugated goat anti-mouse IgG (Fc fraction) was used as a control and is the secondary antibody used for ELISA detection of mouse IgG antibody. It gave values equal to the use of 2% eBSA-PBS alone. Anti-*T. vaginalis* mouse serum has been described previously [29]. The normal mouse serum (NMS) and anti-AEG::SOE2 mouse serum are as described in Figure 4. MAb ALD30A and the MAb cocktail are to the epitopes of the recombinant AEG::SOE2 (Figure 1). MAb L64 is an irrelevant control antibody that reacts with a cytoplasmic protein of *T. vaginalis* and has been used previously [22,26]. All MAbs are the same IgG_1_ isotype. **^‡^** Absorbance values were obtained at 405nm. The mean and standard deviation (SD) were calculated for the average of ELISA readings, and all experiments were performed using quadruplicate wells. **^‡‡^** ND, not done.
